# Effect of common pregnancy and perinatal complications on offspring metabolic traits across the life course: a multi-cohort study

**DOI:** 10.1186/s12916-022-02711-8

**Published:** 2023-01-18

**Authors:** Ahmed Elhakeem, Justiina Ronkainen, Toby Mansell, Katherine Lange, Tuija M. Mikkola, Binisha H. Mishra, Rama J. Wahab, Tim Cadman, Tiffany Yang, David Burgner, Johan G. Eriksson, Marjo-Riitta Järvelin, Romy Gaillard, Vincent W. V. Jaddoe, Terho Lehtimäki, Olli T. Raitakari, Richard Saffery, Melissa Wake, John Wright, Sylvain Sebert, Deborah A. Lawlor

**Affiliations:** 1 MRC Integrative Epidemiology Unit at the University of Bristol, Bristol, UK; 2 Population Health Science, Bristol Medical School, University of Bristol, Bristol, UK; 3 Research Unit of Population Health, Faculty of Medicine, University of Oulu, Oulu, Finland; 4 Murdoch Children’s Research Institute, Parkville, VIC Australia; 5 Department of Paediatrics, University of Melbourne, Parkville, VIC Australia; 6 Folkhälsan Research Center, Helsinki, Finland; 7 Clinicum, Faculty of Medicine, University of Helsinki, Helsinki, Finland; 8 Department of Clinical Chemistry, Faculty of Medicine and Health Technology, Tampere University, Tampere, Finland; 9 Finnish Cardiovascular Research Center Tampere, Faculty of Medicine and Health Technology, Tampere University, Tampere, Finland; 10 Department of Clinical Chemistry, Fimlab Laboratories, Tampere, Finland; 11 Department of Paediatrics, Erasmus MC-University Medical Centre Rotterdam, Rotterdam, Netherlands; 12 The Generation R Study Group, Erasmus MC, University Medical Centre, Rotterdam, Netherlands; 13 Section of Epidemiology, Department of Public Health, University of Copenhagen, Copenhagen, Denmark; 14 Bradford Institute for Health Research, Bradford Teaching Hospitals National Health Service Foundation Trust, Bradford, UK; 15 Department of Paediatrics, Monash University, Clayton, VIC Australia; 16 Department of General Practice and Primary Health Care, University of Helsinki and Helsinki University Hospital, Helsinki, Finland; 17 Obstetrics & Gynecology, Yong Loo Lin School of Medicine, National University of Singapore and National University Health System, Singapore, Singapore; 18 Singapore Institute for Clinical Sciences (SICS), Agency for Science and Technology (A*STAR), Singapore, Singapore; 19 Department of Epidemiology and Biostatistics, School of Public Health, Imperial College London, London, UK; 20 Research Centre of Applied and Preventive Cardiovascular Medicine, University of Turku, Turku, Finland; 21 Department of Clinical Physiology and Nuclear Medicine, Turku University Hospital, Turku, Finland; 22 Centre for Population Health Research, University of Turku and Turku University Hospital, Turku, Finland; 23 Liggins Institute, University of Auckland, Auckland, New Zealand; 24 NIHR Bristol Biomedical Research Centre, Bristol, UK

**Keywords:** Life course, Metabolomics, Cohort

## Abstract

**Background:**

Common pregnancy and perinatal complications are associated with offspring cardiometabolic risk factors. These complications may influence multiple metabolic traits in the offspring and these associations might differ with offspring age.

**Methods:**

We used data from eight population-based cohort studies to examine and compare associations of pre-eclampsia (PE), gestational hypertension (GH), gestational diabetes (GD), preterm birth (PTB), small (SGA) and large (LGA) for gestational age (vs. appropriate size for gestational age (AGA)) with up to 167 plasma/serum-based nuclear magnetic resonance-derived metabolic traits encompassing lipids, lipoproteins, fatty acids, amino acids, ketones, glycerides/phospholipids, glycolysis, fluid balance, and inflammation. Confounder-adjusted regression models were used to examine associations (adjusted for maternal education, parity age at pregnancy, ethnicity, pre/early pregnancy body mass index and smoking, and offspring sex and age at metabolic trait assessment), and results were combined using meta-analysis by five age categories representing different periods of the offspring life course: neonates (cord blood), infancy (mean ages: 1.1–1.6 years), childhood (4.2–7.5 years); adolescence (12.0–16.0 years), and adulthood (22.0–67.8 years).

**Results:**

Offspring numbers for each age category/analysis varied from 8925 adults (441 PTB) to 1181 infants (135 GD); 48.4% to 60.0% were females. Pregnancy complications (PE, GH, GD) were each associated with up to three metabolic traits in neonates (*P*≤0.001) with some evidence of persistence to older ages. PTB and SGA were associated with 32 and 12 metabolic traits in neonates respectively, which included an adjusted standardised mean difference of −0.89 standard deviation (SD) units for *albumin* with PTB (95% CI: −1.10 to −0.69, *P*=1.3×10^−17^) and −0.41 SD for *total lipids in medium HDL* with SGA (95% CI: −0.56 to −0.25, *P*=2.6×10^−7^), with some evidence of persistence to older ages. LGA was inversely associated with 19 metabolic traits including lower levels of cholesterol, lipoproteins, fatty acids, and amino acids, with associations emerging in adolescence, (e.g. −0.11 SD *total fatty acids*, 95% CI: −0.18 to −0.05, *P*=0.0009), and attenuating with older age across adulthood.

**Conclusions:**

These reassuring findings suggest little evidence of wide-spread and long-term impact of common pregnancy and perinatal complications on offspring metabolic traits, with most associations only observed for newborns rather than older ages, and for perinatal rather than pregnancy complications.

**Supplementary Information:**

The online version contains supplementary material available at 10.1186/s12916-022-02711-8.

## Background

There are widespread changes in maternal circulating metabolites during pregnancy, which return to normal after pregnancy [1]. These alterations are likely to be important for maternal health, normal foetal growth, and development [2]. Maternal metabolic profiles are associated with different common pregnancy and perinatal complications including pre-eclampsia (PE), gestational hypertension (GH), gestational diabetes (GD), preterm birth (PTB) and small (SGA) and large for gestational age (LGA) [3–9]. PE, GH, PTB, and SGA tend to relate to placental pathologies/foetal growth restriction [10, 11], whereas GD and LGA relate to foetal overgrowth [12, 13]. Both foetal growth restriction and overgrowth might have long-lasting metabolic effects, which may in turn increase cardiovascular disease (CVD) risk [14–16].

Studies indicate common pregnancy/perinatal complications associated with cardiovascular disease in the offspring [17–22]. However, to the best of our knowledge, effects of common pregnancy/perinatal complications on offspring metabolic traits, and whether these change with age, have not been examined. Identifying whether effects on offspring metabolism are short lived, persist across life, emerge later, or strengthen/weaken with age can improve our understanding of CVD aetiology and may inform the timing of interventions. Therefore, the aim of this study was to examine associations of common pregnancy and perinatal complications related to foetal growth restriction (PE, GH, PTB, and SGA) and foetal overgrowth (GD, LGA) with targeted metabolomic profiles across the offspring life course and investigate whether associations differ by offspring age at assessment of metabolic traits.

## Methods

This study was carried out by following a pre-specified analysis plan and code developed by AE and DAL (https://osf.io/vfd7g) and is reported in accordance with The Strengthening the Reporting of Observational Studies in Epidemiology (STROBE) Statement guidelines for cohort studies [23].

### Cohort studies

Participating cohorts were recruited from the EU Child Cohort Network (EUCCN) [24]; a consortium of European and Australian pregnancy/birth cohorts. Studies were included if they had data on (i) at least one pregnancy/perinatal complication, (ii) offspring metabolic profiles measured at any age in plasma/serum by high-throughput proton nuclear magnetic resonance (NMR)-based targeted metabolomics platform (the most widely used platform across EUCCN cohorts) [25], and (iii) prespecified confounders.

Eight cohorts were eligible, and all agreed to participate in this analysis. These were the UK-based Avon Longitudinal Study of Parents and Children (ALSPAC) [26–30], Born in Bradford Study (BiB) [31, 32], Finland-based Young Finns Study (YFS) [33–35], Northern Finland Birth Cohort 1966 (NFBC1966) [35–37], Northern Finland Birth Cohort 1986 (NFBC1986) [37], and Helsinki Birth Cohort Study (HBCS) [38], and the Australia-based Barwon Infant Study (BIS) [36, 37] and Longitudinal Study of Australian Children’s Child Health CheckPoint (CheckPoint) [39, 40]. A ninth cohort, the Generation R study from the Netherlands, had metabolic traits measured by mass spectrometry in cord blood and non-fasting blood samples at mean age 9.8 years and was used to replicate results for any overlapping NMR-derived traits [41–43]. More detail on included cohorts is in Additional file 1: Supplemental Methods.

### Ethics, consent, and permissions

All cohorts had ethical approval from their relevant local or national ethics committees and study participants provided informed consent or assent to participate in the respective cohorts and secondary data analyses. Details on ethics approvals and consent for each cohort can be found in the Additional file 1: Supplemental Methods.

### Pregnancy and perinatal complications

Six common pregnancy/perinatal complications related to foetal growth restriction (PE, GH, PTB, and SGA) and foetal overgrowth (GD, LGA) were included. Data on pregnancy/perinatal complications were extracted from medical records or reported in questionnaires. Description of how these were recorded in each cohort is in Additional file 1: Supplemental Methods. Data harmonisation has been previously described [44]. Briefly, PE was defined as elevated blood pressure >20 weeks gestation (≥140 mmHg systolic or ≥90 mmHg diastolic), and proteinuria (>0.3g per 24 h), or by HELLP syndrome (haemolysis, elevated liver enzymes, and low platelets) [45]. GH was defined as new onset hypertension after 20 weeks of gestation, with previously normal blood pressure, without proteinuria or manifestations of PE. GD was defined as glucose intolerance with onset or first diagnosis in pregnancy and continuing past 24–28 weeks of gestation. This was based on a 75-g oral glucose tolerance test comprising fasting and 2h post-load samples at around 26–28 weeks gestation in BiB, and by extraction from health records or questionnaire responses in the other cohorts. PTB was defined as ≤37 completed weeks (or ≤259 days) at birth. SGA and LGA were defined based on the World Health Organisation foetal growth charts, using the 5^th^ and 95^th^ percentiles as cut-offs, respectively [46].

Offspring exposed to PE, GH, PTB, or GD, were compared to those not exposed to the specific complication. Offspring born SGA and LGA, were each compared to those born appropriate size for gestational age (AGA) offspring, i.e. with SGA/LGA excluded in turn.

### Offspring NMR-derived metabolic traits and age categories

A proton NMR-based targeted metabolomics platform [25] was used to quantify up to 250 offspring metabolic traits (including derived variables) in plasma/serum samples in the eight participating cohorts. The NMR platform uses a single experimental setup to simultaneously quantify metabolic traits from each plasma/serum sample. Metabolic traits were quantified in absolute concentration units or ratios and included circulating lipoprotein lipids and subclasses, fatty acids and their compositions, amino acids and traits related to glycolysis, ketone bodies, fluid balance, and an inflammatory marker. The manufacturer’s standard quality control procedures were performed in all cohorts [25].

Traits were analysed using non-fasting samples in cord blood and infancy, semi-fasting samples in childhood and adolescence, and fasting samples at older ages. Differences in fasting status were because younger participants were not asked to fast prior to clinic visits due to compliance/ethical issues. Metabolic trait ratios were excluded because of challenges in their interpretation, leaving up to 167 metabolic traits in the analysis. Description of the methods and ages at the assessment of metabolic traits in each cohort is in Additional file 1: Supplemental Methods. The metabolic traits available in each cohort are listed in Additional file 2: Data Set 1.

Metabolic traits measured at all available ages from each cohort were included. Cohort results were combined into five pre-specified age categories for meta-analysis, chosen to reflect key life course periods and to maximise the number of participants, with results from at least two cohorts available for each age group. The age categories were neonates (cord blood), infancy (mean age 1.1 to 1.6 years), childhood (mean age 4.2 to 7.5 years), adolescence (mean age 12.0 to 16.0 years), and adulthood (mean age 22.0 to 67.8 years).

### Confounders

To estimate unconfounded associations of pregnancy/perinatal complications with offspring metabolic traits, we identified and adjusted for potential confounders, i.e. factors that could plausibly cause pregnancy/perinatal complications and influence offspring metabolism, and avoided adjustment for mediators on causal path of any effect (e.g. offspring adiposity) and other sources of collider bias [47]. The identified confounders were maternal education (the most available and consistent indicator of early life socioeconomic position across cohorts), ethnicity, age at pregnancy/birth of offspring, parity, pre/early pregnancy body mass index (BMI) and smoking in pregnancy. Offspring sex and age at metabolic trait assessment were included as adjustments to improve modelling precision. Details on how confounders were measured in each cohort are provided in Additional file 1: Supplemental Methods. Harmonised variables were derived as described previously [44].

ALSPAC, BiB, BIS, and YFS were able to adjust for all confounders. NFBC1966, NFBC1986, and HBCS did not adjust for ethnicity, but they were predominantly white ethnicity, HBCS was unable to adjust for smoking, and CheckPoint was unable to adjust for BMI or parity.

### Statistical analysis

Associations between each of the six pregnancy/perinatal complications and each offspring NMR-derived metabolic trait from all available timepoints were examined in each cohort by fitting adjusted (for confounders plus offspring age and sex) linear regression models (with robust standard errors). Analyses were restricted to those with complete data on the relevant pregnancy/perinatal complication, metabolic trait, and confounders. The impact of missing data was explored by comparing the characteristics of included offspring with those that were excluded due to missing data (Additional file 3: Table S1). To allow comparison of results across different pregnancy/perinatal complications, traits, and ages, metabolic traits were analysed using cohort-specific standard deviation (SD) units (mean=0, SD=1).

Cohort-specific results were then combined using meta-analysis in five age categories for neonates, infancy, childhood, adolescence, and adulthood, using a random effects model to allow for between-cohort heterogeneity. Variability in the meta-analysis results that were due to between-cohort heterogeneity was measured by computing the *I*^*2*^ statistic [48, 49]. Where evidence of substantial between-cohort heterogeneity was found, we inspected each cohort’s results to identify the reason for heterogeneity. A *P*-value threshold of *P*≤0.001 was selected to identify statistically robust associations between the pregnancy/perinatal complications and metabolic traits. This was chosen instead of a Bonferroni-corrected *P*-value because many metabolic traits are highly correlated [40] and so independent tests were not performed, yet our threshold is still more stringent than a conventional *P*-value threshold. For association that reached this threshold in one age category, we highlighted the equivalent association in all other age categories, to explore changes with age in the context of different numbers of participants for each age category (Table 1).

**Table 1:** Characteristics of the cohorts and offspring included in the analysis^a^

*Cohort name*	ALSPAC	BiB	YFS	NFBC1986	NFBC1966	HBCS	BIS	CheckPoint
*Cohort country*	UK	UK	Finland	Finland	Finland	Finland	Australia	Australia
*Offspring birth years*	1990–1992	2007–2011	1962–1977	1985–1986	1966	1934–1944	2010–2013	2003–2004
*Mean age(s) (in years) at assessment of NMR-derived metabolic traits*	7.5 years, 15.4 years, 17.8 years, 24.5 years	0 (cord blood), 1.6 years	22.0 years	16.0 years	31.2 years, 46.6 years	67.8 years	0 (cord blood), 1.1 years, 4.2 years	12.0 years
*Sex [No. (%)]*								
Male	3216 (49.6)	1290 (51.6)	204 (40.0%)	2305 (50.0)	2400 (47.9)	466 (44.0%)	374 (51.5)	429 (47.8)
Female	3263 (50.4)	1209 (48.4)	305 (60.0%)	2309 (50.0)	2607 (52.1)	593 (56.0%)	352 (48.5)	469 (52.2)
*Pre-eclampsia [No. (%)]*								
No	6335 (98.1)	2328 (97.2)	499 (98.0%)	3596 (97.5)	2883 (96.3)	-	701 (97.0)	-
Yes	120 (1.9)	66 (2.8)	10 (2.0%)	91 (2.5)	110 (3.7)	-	22 (3.0)	-
*Gestational hypertension [No. (%)]*								
No	5433 (85.8)	2215 (92.5)	492 (97.0%)	3596 (96.4)	2883 (86.9)	-	713 (98.2)	843 (94.2)
Yes	902 (14.2)	179 (7.5)	17 (3.0%)	134 (3.6)	406 (13.1)	-	13 (1.8)	52 (5.8)
*Gestational diabetes [No. (%)]*								
No	6424 (99.5)	1091 (84.7)	468 (92%)	-	-	-	582 (95.7)	846 (95.1)
Yes	31 (0.5)	197 (15.3)	41 (8%)	-	-	-	26 (4.3)	44 (4.9)
*Preterm birth [No. (%)]*								
No	6156 (95.0)	2342 (93.7)	460 (90,0%)	4381 (95.0)	4775 (95.4)	1000 (94.4)	682 (93.9)	800 (89.6)
Yes	323 (5.0)	157 (6.3)	49 (10.0%)	230 (5.0)	225 (4.6)	59 (5.6)	44 (6.0)	93 (10.4)
*Small for gestational age [No. (%)]*								
No (appropriate size for age)	5446 (94.4)	2140 (90.8)	-	3842 (96.5)	3928 (94.5)	-	631 (98.3)	764 (95.3)
Yes	323 (5.6)	216 (9.2)	-	138 (3.5)	227 (5.6)	-	11 (1.7)	38 (4.7)
*Large for gestational age [No. (%)]*								
No (appropriate size for age)	5446 (89.9)	2140 (93.8)	-	3842 (86.1)	3928 (89.6)	-	631 (88.3)	764 (89.6)
Yes	610 (10.1)	142 (6.2)	-	621 (13.9)	454 (10.4)	-	84 (11.8)	89 (10.4)

A total of 24,864 offspring from 8 cohorts were included in this study for the sub-group meta-analysis

^a^ Characteristics presented for study participants with data on at least 1 pregnancy/perinatal complication, NMR-derived metabolic traits, and all available confounders. For the cohort studies that had metabolic traits measured at multiple timepoints, the table shows the characteristics for the study participants with data from the timepoint with the largest sample size

We further investigated the change in associations with older age by fitting confounder-adjusted natural cubic spline mixed effects trajectory models [50] in 4980 ALSPAC offspring with up to 4 repeated NMR-based assessments from 7 to 26 years. This analysis included all offspring with complete data on the relevant pregnancy/perinatal complication and confounders, and at least one of the 4 repeated measures of metabolic traits (i.e. those with incomplete outcome measurements were included). Because ALSPAC data spans ages 7–26 years, trajectory analysis was only done for the metabolic traits showing a meta-analysis association in childhood, adolescence, or adulthood (and not in neonates or infancy). An interaction between the pregnancy/perinatal complication and age was included to allow different trajectories for exposed/nonexposed offspring. The predicted mean trajectories and differences in metabolic traits were obtained [51].

Lastly, we sought support for associations identified with NMR-derived traits by performing a replication analysis in the Generation R study. Replication was done for all NMR-derived traits that were available from the mass spectrometry platform, using regression models with similar adjustment for confounders.

## Results

A total of 24,864 offspring from 8 cohorts were included in this study for the age sub-group meta-analysis (Table 1). Offspring numbers in each age category analysis varied from 8925 adults (441 PTB) to 1181 infants (135 GD) (Table 2). Offspring birth years were from 1934 to 2013, and 48.4% to 60.0% were female (Table 1). The proportion of offspring exposed to pregnancy/perinatal complications in each cohort ranged from 1.9% to 3.7% PE, 1.5% to 14.2% GH, 0.5% to 15.3% GD, 4.6% to 10.4% PTB, 3.5% to 9.2% SGA, and 6.2% to 13.9% LGA (Table 1). Those excluded due to missing data had lower maternal education, younger maternal age, and higher prevalence of maternal pregnancy smoking and non-white ethnicity than those included in the analysis (Additional file 3: Table S1).

**Table 2:** Number of cohorts, offspring, and NMR-derived metabolic traits included in each life course stage analysis

Age category	Pregnancy and perinatal complications					
	PE	GH	*GD*	*PTB*	*SGA*	*LGA*
*Neonate*^a^						
N-exposed/total (N-cohorts/traits)	89/3117 (2/76)	192/3120 (2/76)	223/1896 (2/76)	201/3225 (2/76)	227/2998 (2/76)	226/2997 (2/76)
*Infancy*^b^						
N-exposed/total (N-cohorts/traits)	53/1894 (2/135)	101/1897 (2/135)	135/1181 (2/135)	156/1963 (2/135)	141/1831 (2/135)	132/1822 (2/135)
*Childhood*^c^						
N-exposed/total (N-cohorts/traits)	130/6883 (2/148)	910/6774 (2/148)	45/6806 (2/148)	350/6908 (2/148)	331/6144 (2/148)	664/6447 (2/148)
*Adolescence*^d^						
N-exposed/total (N-cohorts/traits)	134/6117 (2/162)	538/7012 (3/162)	57/3320 (2/162)	429/7947 (3/162)	466/7137 (3/162)	943/7614 (3/162)
*Adulthood*^*e*^						
N-exposed/total (N-cohorts/traits)	154/5850 (3/156)	760/4135 (3/156)	50/2857 (2/156)	441/8925 (4/155)	313/6387 (2/160)	652/6727 (2/160)

*PE* pre-eclampsia, *GH* gestational hypertension, *GD* gestational diabetes, *PTB* preterm birth, *SGA* small for gestational age, *LGA* large for gestational age

^a^ Metabolic traits were assessed in cord blood in the BiB cohort (*n*=2499 offspring) and the BIS cohort (*n*=726 offspring)

^b^ NMR-derived metabolic traits were assessed at mean age 1.1 years (SD=0.1) in the BIS cohort (*n*=591) and at mean age 1.6 years (standard deviation (SD)=0.5) in the BiB cohort (*n*=1373)

^c^ NMR-derived metabolic traits were assessed at mean age 4.2 years (SD=0.3) in the BIS cohort (*n*=429) and at mean age 7.5 years (SD=0.2) in the ALSPAC cohort (*n*=6206)

^d^ NMR-derived metabolic traits were assessed at mean age 12.0 years (SD=0.4) in the CheckPoint cohort (*n*=898), at mean age 15.4 years (SD=0.3) in the ALSPAC cohort (*n*=2348), and at mean age 16.0 years (SD=0.4) in the NFBC1986 cohort (*n*=4614)

^e^ NMR-derived metabolic traits were assessed at mean age 22.0 years (SD=7.0) in the YFS cohort (SD=*n*=509), at mean age 24.5 years (SD=0.8) in the ALSPAC cohort (*n*=2256), at mean age 46.6 years (SD=0.6) in the NFBC1966 cohort (*n*=5137), and at mean age 67.8 years (SD=4.4) in the HBCS cohort (*n*=1057). Adult meta-analysis was done using NFBC1966 age 46.6 years results because of the bigger sample size at this; meta-analysis results did not differ when repeated using NFBC1966 age 31.2 years and so only the age 46 results are included

### Pre-eclampsia, gestational hypertension, preterm birth and small for gestational age associations with offspring metabolic traits

**Fig. 1:**
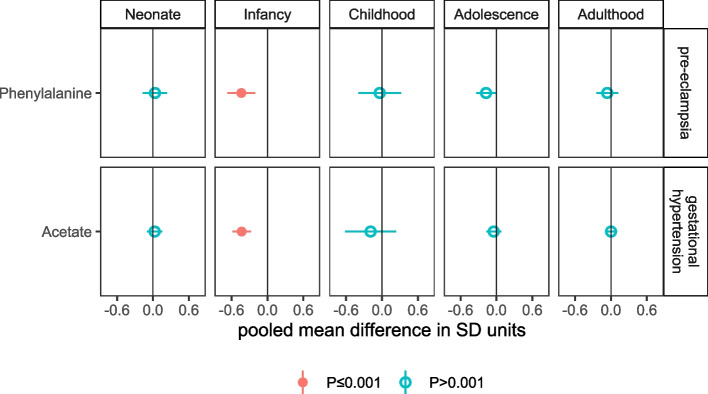
Figure shows the pooled adjusted mean differences in standard deviation (SD) units in offspring NMR-derived metabolic traits for pre-eclampsia (minus no pre-eclampsia) and gestational hypertension (minus no gestational hypertension), for associations reaching the *P*<0.001 threshold in any one of the five age categories, and equivalent associations in all other age categories (to explore differences by age). Results are adjusted for offspring sex age, and confounders (maternal education, parity age at pregnancy, ethnicity, pre/early pregnancy BMI and smoking). Horizontal bars represent the 95% confidence intervals. Numerical values of these differences are presented in Table S2

PE, GH, PTB, and SGA were associated with 1, 1, 32, and 12 metabolic traits, respectively, with all but 3 of these associations observed for neonates only. PE was associated with lower levels of aromatic amino acid *phenylalanine* in infancy (mean difference: −0.44 SD, 95% CI: −0.67 to −0.22, *P*=0.0002), with differences close to zero in neonates, children, and adults, but there was some evidence in favour of lower levels in adolescents (mean difference: −0.17 SD, 95% CI: −0.34 to −0.01, *P*=0.03) (Fig. 1, Additional file 4: Table S2). GH was inversely associated with the ketone *acetate* in infants (mean difference: −0.43 SD, 95% CI: −0.59 to −0.28, *P*=3.7×10^−8^), although equivalent differences for other age groups were either close to zero or were imprecisely estimated (Fig. 1, Additional file 4: Table S2).

**Fig. 2:**
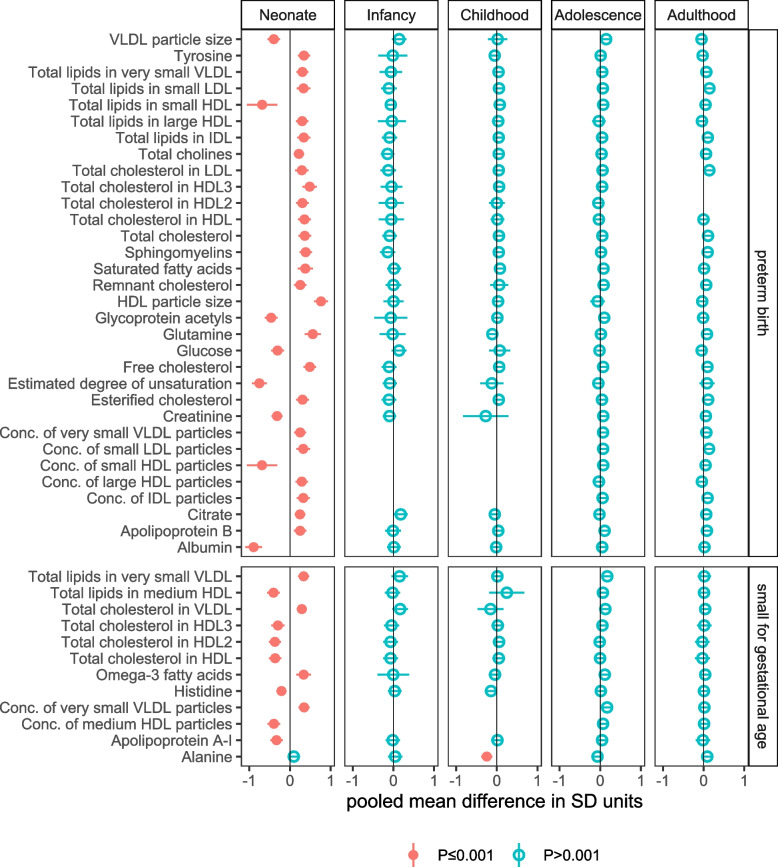
Figure shows the pooled adjusted mean differences in standard deviation (SD) units in offspring NMR-derived metabolic traits for preterm birth (minus not preterm birth), and small for gestational age (minus appropriate size for gestational age), for associations reaching the *P*<0.001 threshold in any one of the five age categories, and equivalent associations in all other age categories (to explore differences by age). Results are adjusted for offspring sex age, and confounders (maternal education, parity age at pregnancy, ethnicity, pre/early pregnancy BMI and smoking). Horizontal bars represent the 95% confidence intervals. Numerical values of these differences are presented in Table S2

PTB was inversely associated with *total lipids in small HDL*, *concentration of small HDL particles*, *degree of unsaturation*, *glucose*, fluid balance markers *creatinine* and *albumin*, and inflammatory marker *glycoprotein acetyls* (*GlycA*), and positively associated with cholesterol measures, lipoprotein subclasses, glycerides, phospholipids, *Apolipoprotein B*, *saturated fatty acids*, the α-amino acid *glutamine*, and the aromatic amino acid *tyrosine*, with all associations observed in neonates (Fig. 2). Associations with neonate metabolic traits for PTB (vs. not PTB) ranged in magnitude from 0.22 SD (95% CI: 0.09 to 0.35, *P*=0. 0007) for *total cholines* to −0.89 SD (95% CI: −1.10 to −0.69, *P*=1.3×10^−17^) for *albumin*. For most results, equivalent differences across older age categories were close to zero though there was some evidence of persistence to older ages for some metabolic traits, e.g. the mean difference in *total lipids in small LDL* in neonates and adults was 0.33 SD (95% CI: 0.16 to 0.50, *P*=0.0001), and 0.14 SD (95% CI: 0.04 to 0.24, *P*=0.004), respectively (Fig. 2, Additional file 4: Table S2).

SGA (vs. AGA) in neonates was inversely associated *with total cholesterol in HDL*, *total cholesterol in HDL2*, *total cholesterol in HDL3*, *total lipids in medium HDL*, *concentration of medium HDL particles*, *apolipoprotein A-I*, and *histidine*, and positively associated with *total cholesterol in VLDL*, *total lipids in very small VLDL*, *concentration of very small VLDL particles*, and *omega-3 fatty acids* (Fig. 2). Differences ranged in magnitude from −0.21 SD (95% CI: −0.33 to −0.08, *P*=0.001) for *histidine* to −0.41 SD (95% CI: −0.56 to −0.25, *P*=2.6×10^−7^) for *total lipids in medium HDL* (Additional file 4: Table S2). Most were reduced at older ages but there was some evidence for higher levels of *total cholesterol in VLDL*, *total lipids in very small VLDL*, *concentration of very small VLDL particles*, and *omega-3 fatty acids* during adolescence, e.g. mean differences in *total lipids in very small VLDL* in neonates and adolescence were 0.34 SD (95% CI: 0.20 to 0.47, *P*=1.4×10^−6^), and 0.17 SD (95% CI: 0.06 to 0.29, *P*=0.003), respectively. SGA was also inversely associated with amino acid *alanine* in childhood (−0.25 SD, 95% CI: −0.38 to −0.11, *P*=0.0003), with no clear differences in *alanine* at other age groups (Fig. 2).

Of the NMR-derived metabolic traits that PE, GH, PTB, and SGA were associated with, four were found among mass spectroscopy measures in the Generation R Study and included for replication (PTB: *tyrosine*, *sphingomyelins*, *glutamine*; SGA: *histidine*). Consistent with the pooled difference for PTB neonates in NMR-derived *tyrosine* (0.34 SD, 95% CI: 0.19 to 0.49, *P*=7.0×10^−6^) and *sphingomyelins* (0.39 SD, 95% CI: 0.23 to 0.54, *P*=6.8×10^−7^), PTB was also associated with higher *tyrosine* (0.82 SD, 95% CI: 0.40 to 1.24, *P*=0.0001, *n*=725 (29 PTB)) and *sphingomyelins* (0.49 SD, 95% CI: 0.07 to 0.90, *P*=0.02) in neonates in Generation R. In contrast, the meta-analysis association of PTB with *glutamine* (0.56 SD, 95% CI: 0.36 to 0.76, *P*=2.4×10^−8^) did not replicate in Generation R (−0.09, 95% CI: −0.51 to 0.34, *P*=0.7). Lastly, the mean difference in *histidine* for SGA (vs. AGA) neonates was similar in the meta-analysis (−0.21 SD, 95% CI: −0.33 to −0.08, *P*=0.001) and Generation R but this result was imprecisely estimated (−0.25 SD, 95% CI: −0.61 to 0.11, *P*=0.2, *n*=651 (35 SGA)).

### Gestational diabetes and large for gestational age associations with offspring metabolic traits

**Fig. 3:**
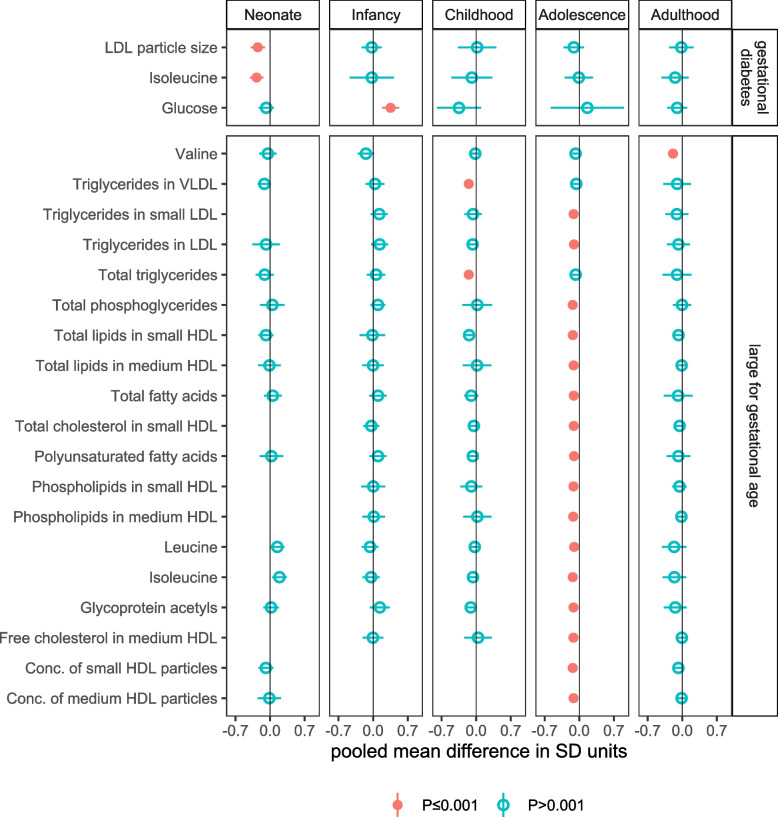
Figure shows the pooled adjusted mean differences in standard deviation (SD) units in offspring NMR-derived metabolic traits for gestational diabetes (minus no gestational diabetes), and for large gestational age (minus appropriate size for gestational age), for associations reaching the *P*<0.001 threshold in any one of the five age categories, and equivalent associations in all other age categories (to explore differences by age). Results are adjusted for offspring sex age, and confounders (maternal education, parity age at pregnancy, ethnicity, pre/early pregnancy BMI and smoking). Horizontal bars represent the 95% confidence intervals. Numerical values of these differences are presented in Table S2

GD and LGA were associated with 3 and 19 metabolic traits, respectively. GD was associated with smaller *LDL particle size* (mean difference: −0.25 SD, 95% CI: −0.39 to −0.10, *P*=0.0007) and with lower *isoleucine* (mean difference: −0.27 SD, 95% CI: −0.41 to −0.14, *P*=0.00008) in neonates, with differences in both metabolic traits close to zero for older ages (Fig. 3). GD was positively associated with *glucose* in infants (mean difference: 0.35 SD, 95% CI: 0.18 to 0.52, *P*=0.00005), with no difference in *glucose* found for other age categories (Fig. 3).

Of the nineteen associations of LGA with offspring metabolic traits, none were observed in neonates or infants, two were observed in children, sixteen in adolescents, and one in adults (Fig. 3). All were inverse associations and represented lower levels of cholesterol, fatty acids, lipoprotein subclasses, three branched-chain amino acids, and the inflammatory marker *glycoprotein acetyls* for LGA (vs. AGA). Associations ranged in magnitude from −0.10 SD (95% CI: −0.16 to −0.04, *P*=0.001) for *leucine* in adolescents to −0.19 SD (95% CI: −0.29 to −0.09, *P*=0.0003) for *valine* in adulthood (Additional file 4: Table S2). For associations seen in adolescence, equivalent associations in adults were slightly attenuated and had wider confidence intervals, and for some traits, there was also evidence for lower levels in childhood, e.g. difference in *total lipids in small HDL* in childhood, adolescence and adulthood was −0.14 SD (95% CI: −0.24 to −0.04, *P*=0.004), −0.13 SD (95% CI: −0.19 to −0.07, *P*=.00006), and −0.08 SD (95% CI: −0.16 to 0.01, *P*=0.1), respectively.

Of the 22 associations identified for GD and LGA, only one metabolic trait overlapped with mass spectroscopy measures in the Generation R Study (LGA and child *total triglycerides*). The inverse association in our meta-analysis (difference in child *total triglycerides* for LGA vs. AGA (−0.15 SD, 95% CI: −0.24 to −0.06, *P*=0.001) was weaker and imprecisely estimated in Generation R (−0.04 SD, 95% CI: −0.40 to 0.32, *P*=0.8, n=339 total with *n*=75 LGA),

### Overlap in metabolic trait associations

Comparing identified pregnancy/perinatal complication–metabolic trait associations showed that PE/GH were each associated with a different metabolic trait, PTB/SGA were associated with *total HDL cholesterol, total HDL2 cholesterol, and total HDL3 cholesterol* in neonates (higher with PTB and lower with SGA), whereas GD (neonate) and LGA (adolescent) were inversely associated with *isoleucine*. Additionally, SGA (neonates) and LGA (adolescents) were both inversely associated with *concentration of medium HDL particles*, PTB (neonate) and LGA (adolescence) were both inversely associated with *GlycA* and *total lipids in small HDL*, and both PTB (inverse association: neonates) and GD (positive association: infants) were associated with *glucose*.

### Between-cohort differences

**Fig. 4:**
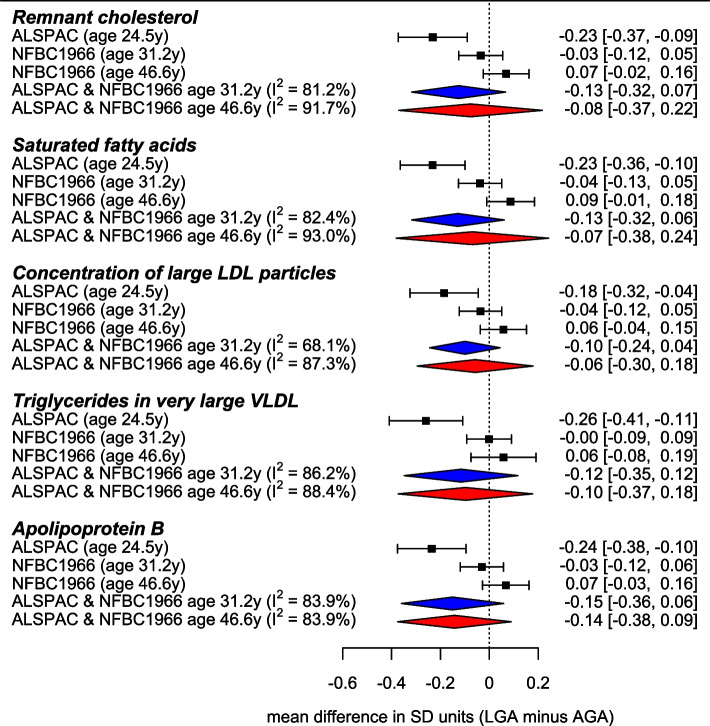
Figure shows the cohort-specific and pooled adjusted mean differences in standard deviation (SD) units in five NMR-derived metabolic traits between adults born large gestational age (LGA) and appropriate size for gestational age (AGA). The pooled results from ALSPAC and NFBC19666 (age 46.6 years) from the meta-analysis are presented, with the pooled result for ALSPAC and age 31.2 years NFBC19666 also presented to highlight differences with age

For most (72%) of the total 3787 results there was little to no between-cohort heterogeneity (*I*^2^≤25%, with *I*^2^=0% for 86% of these), and 8% of the results showed evidence of substantial or high heterogeneity between cohorts (*I*^2^≥75%) (Additional file 5: Data Set 2). Four of these were results that met the *P*≤0.001 threshold and all were for PTB neonates (total lipids in small and very large HDL, and concentrations of small and very large HDL particles). Inspecting results from the cohorts contributing to this age (BiB and BIS) revealed a consistent direction of association in both but mean differences in BiB double those in BIS (e.g. mean difference in *total lipids in small HDL* was −0.86 SD in BiB and −0.48 SD in BIS. Among other results with substantial or high heterogeneity, 97 were for LGA–adult metabolic traits. Further investigation revealed that this was because LGA was inversely associated with adult biomarkers in ALSPAC (age 24.5 years), with smaller positive associations seen in NFBC1966 (age 46.6 years). Notably, results for NFBC1966 at age 31.2 years were between the ALSPAC age 24.5 years results and the NFBC1966 age 46.6 years results, suggesting a possible age effect (Fig. 4).

### ALSPAC age-change trajectory analysis

**Fig. 5:**
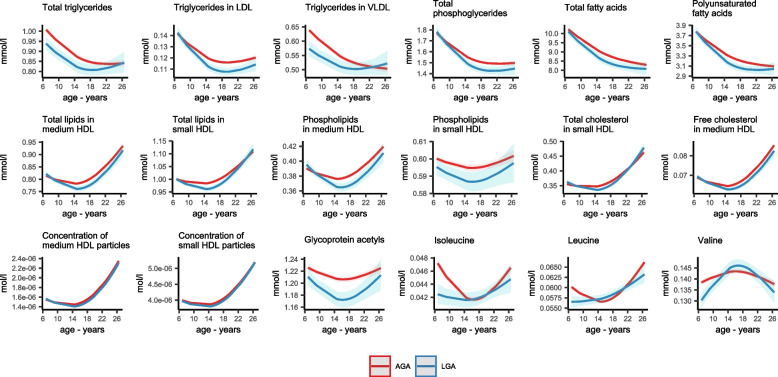
Figure shows the predicted mean NMR-derived metabolic trait trajectories from age 7–26 years for ALSPAC offspring born large for gestational age (LGA, *N*=500) and appropriate size for gestational age (AGA, *N*=4480), for 18 metabolic traits that were identified in the meta-analysis. Predicted values were obtained from adjusted (for sex and confounders) natural cubic spline mixed effects models that included an interaction term with age to allow LGA and AGA groups to have different mean metabolic trait trajectories. Predicted mean differences are presented in Figure S2

Of the twenty associations identified with metabolic traits beyond infancy, one was for SGA, nineteen were for LGA, and all except one for LGA were included in trajectory analysis from childhood to adulthood in the ALSPAC cohort. Consistent with the meta-analysis, SGA (vs. AGA) was associated with lower *alanine* at age 7 years, and this difference was reduced with increasing child age, with *alanine* slightly higher in SGA from mid adolescence (Additional file 6: Figure S1). Similarly, most associations between LGA and metabolic traits appeared to change with age from childhood to adulthood e.g. the inverse association in the meta-analysis between LGA (vs. AGA) and *triglycerides in VLDL* in adolescence was attenuated with older age (Fig. 5, Additional file 7: Figure S2).

## Discussion

We examined the association between common pregnancy/perinatal complications and NMR-derived metabolic profile across the offspring life course in eight population-based cohort studies. Pregnancy complications (PE, GH, GD) were related to only a few metabolic traits mostly in neonates, with little evidence of persistence. More extensive disruptions in neonate metabolic traits were seen for perinatal complications, mostly for PTB, with little evidence of persistence to older ages, except for differences in metabolic traits with LGA, which mostly emerged during adolescence and appeared to weaken by older adulthood.

To the best of our knowledge, ours is the first study to simultaneously investigate short- and long-term effects of common pregnancy/perinatal complications on offspring metabolomics. We found that most pregnancy/perinatal complications were associated with metabolic traits in neonates only with little evidence of persistence of associations beyond early life. This is somewhat in contradiction to commonly expressed views on the importance of the pregnancy period for offspring long-term health. However, our results are consistent with Mendelian randomisation studies that show that intrauterine exposures related to variation in birth weight are unlikely to causally influence offspring metabolic health [53, 54]. Most of the associations with metabolic traits in our study were observed in preterm compared with term/post-term born neonates, which appears to support earlier findings of widespread gestational age-dependent effects on metabolites from an untargeted metabolomics analysis in 298 neonates [52].

Our finding of inverse associations of LGA with adolescent metabolic traits is consistent with a study of 18,288 adolescents and adults with the same metabolomics platform as our study [55] which showed a higher mean birth weight across the distribution was associated with healthier lipid profile, including lower triglycerides, and other lipids that we see inverse associations of LGA within our study. These findings may reflect an interplay with offspring adiposity and puberty whereby LGA offspring have higher prepubertal body fat compared to AGA [56] with this leading to earlier puberty for LGA [57, 58]. Earlier puberty in turn might influence metabolic traits [59] and explain these findings. For example, a decreasing body fat with advanced puberty [60] in the earlier maturing LGA adolescents might explain their healthier metabolic profile at this age [61]. This could also explain why the association of LGA with healthier metabolic profile in adolescence was weakened or even reversed by early midlife (because all other offspring would have already completed puberty). Shared lifestyles between the mothers and offspring might also contribute to these findings [62, 63].

Strengths of this work include the larger sample size compared with previous studies and the examination of metabolic traits during important life periods including whether these change over the life course. Limitations include the small numbers of exposed offspring despite the relatively larger sample size, (e.g. 18.4% exposed to GH — the most common complication). This also meant that we had little power (and so were unable) to examine sex interactions or explore associations separately by sex. Residual confounding due to use of crude harmonised variables across cohorts [64, 65] and unmeasured confounders (e.g. maternal health) could influence our findings. Complete case analysis may have reduced precision of estimates and introduced bias due to missing data and limited the generalisability of our findings due to the difference in characteristics between included and excluded participants. Bias due to missing data on metabolic traits is reduced in the ALSPAC trajectory analysis because all offspring with incomplete outcome measures were included, under the missing at random assumption (i.e. the probability of a missing outcome measurement depends on the observed values of the outcome, conditional on the covariates in the model).

Attrition could introduce a selection bias which may contribute to null/weaker associations in adults and reduced sensitivity to detect associations with small effect sizes, if older offspring were healthier (with a lower prevalence of complications) than those lost to follow-up. The *P*-value threshold used to identify robust associations was somewhat arbitrary, so results require replication in other studies. Approaches to select an effective number of tests have been developed [66] but to the best of our knowledge have not been extended to multi-cohort analyses, like our study. We have provided all coefficients and exact *P*-values, which readers can use to apply their own *P*-value thresholds (Additional file 5: Data Set 2). Only one of five cohorts included in the GD analysis had universal diagnostic testing at the time of pregnancy therefore, some misclassification of GD is possible. Direct measurements of neonatal body fat were not examined and may have provided greater insight than birthweight-derived exposures (SGA and LGA). Finally, there was a wide age gap for adults, especially for preterm birth where 67-year-olds were included, which might limit the interpretation of results in adults.

## Conclusions

Our results offer reassurance that PE, GH, and GD do not result in widespread metabolic disruption in offspring and that more widespread disruptions for PTB and SGA are mostly confined to neonates. Differences in offspring metabolic traits for LGA require further exploration to establish why they primarily arose during adolescence.

## Supplementary Information

**Additional file 1: Supplemental Methods**. Description of the included cohorts and measurements.

**Additional file 2: Data Set 1.** List of all available offspring NMR-derived metabolic traits in each cohort.

**Additional file 3: Table S1.** Comparison of included participants with those excluded due to missing data from the ALSPAC cohort.

**Additional file 4: Tables S2.** Association of pregnancy and perinatal complications with offspring NMR-derived metabolic traits.

**Additional file 5: Data Set 2.** Complete meta-analysis results i.e., the adjusted pooled mean differences in SD units for all NMR-derived metabolic trait at each age category and by each pregnancy/perinatal complication.

**Additional file 6: Figure S1.** Predicted mean Alanine trajectories from age 7-26 years and predicted mean differences in ALSPAC offspring born small for gestational age and appropriate size for gestational age.

**Additional file 7: Figure S2.** Predicted mean differences in NMR-derived metabolic traits for born large for gestational age and appropriate size for gestational age offspring from the ALSPAC cohort.

**Additional file 8: Table S3.** Cohort-specific acknowledgements and funding statements.

## Data Availability

The datasets used during the current study are available to researchers by request from each cohort.

## Abbreviations

AGA: Appropriate size for gestational age
CVD: Cardiovascular disease
EUCCN: EU Child Cohort Network
GD: Gestational diabetes
GH: Gestational hypertension
LGA: Large for gestational age
NMR: Nuclear Magnetic Resonance
PE: Pre-eclampsia
PTB: Preterm birth
SGA: Small for gestational age
