# Supplementary material for: Effect of common pregnancy and perinatal complications on offspring metabolic traits across the life course: a multi-cohort study
Source: BMC Med. 2023 Jan 18;21:23. doi: 10.1186/s12916-022-02711-8 (PMC9850719; doi:10.1186/s12916-022-02711-8)
Supplement: Supplementary file 3 — Additional file 3: Table S1. Comparison of included participants with those excluded due to missing data from the ALSPAC cohort. [file 12916_2022_2711_MOESM3_ESM.docx]

| **Additional file 3: Table S1** Comparison of included participants with those excluded due to missing data from the ALSPAC cohort | | |
| --- | --- | --- |
|  | Included | Excluded |
| **ALSPAC** | N=6479 | N=6772 |
| Maternal age – years [mean (SD)] | 29.7 (4.5) | 27.7 (5.0) |
| Maternal BMI – kg/m^2^ [mean (SD)] | 22.9 (3.7) | 23.0 (4.2) |
|  |  |  |
| Maternal parity [No. (%)] |  |  |
| 0 | 2964 (45.8) | 1813 (44.7) |
| 1 | 2356 (36.4) | 1307 (32.2) |
| 2 or more | 1159 (17.9) | 938 (23.1) |
|  |  |  |
| Maternal ethnicity [No. (%)] |  |  |
| White | 6405 (98.9) | 3135 (96.3) |
| Non-white | 74 (1.1) | 122 (3.8) |
|  |  |  |
| Maternal education [No. (%)] |  |  |
| High | 1102 (17.0) | 246 (7.2) |
| Medium | 4602 (71.0) | 2060 (60.4) |
| Low | 775 (12.0) | 1107 (32.4) |
|  |  |  |
| Maternal smoking [No. (%)] |  |  |
| None | 5208 (80.4) | 1336 (61.7) |
| < 10 per day | 840 (13.0) | 456 (21.1) |
| ≥ 10 per day | 431 (6.7) | 373 (17.2) |

Data shows maternal characteristics for those included in the analysis (i.e., with complete data on the relevant pregnancy/perinatal complication, metabolic trait, and confounders) and those excluded due to missing data on pregnancy/perinatal complications and metabolic traits, and incomplete data on confounders.
