# Supplementary material for: Effect of common pregnancy and perinatal complications on offspring metabolic traits across the life course: a multi-cohort study
Source: BMC Med. 2023 Jan 18;21:23. doi: 10.1186/s12916-022-02711-8 (PMC9850719; doi:10.1186/s12916-022-02711-8)
Supplement: Supplementary file 4 — Additional file 4: Tables S2. Association of pregnancy and perinatal complications with offspring NMR-derived metabolic traits. [file 12916_2022_2711_MOESM4_ESM.docx]

# **Additional file 4: Tables S2** Association of pregnancy and perinatal complications with offspring NMR-derived metabolic traits

|  | **Neonate** | | **Infancy** | | **Childhood** | | **Adolescence** | | **Adulthood** | |
| --- | --- | --- | --- | --- | --- | --- | --- | --- | --- | --- |
|  | Estimate (95%CI) | *P* | Estimate (95%CI) | *P* | Estimate (95%CI) | *P* | Estimate (95%CI) | *P* | Estimate (95%CI) | *P* |
| ***Pre-eclampsia*** |  |  |  |  |  |  |  |  |  |  |
| Phenylalanine | 0.03 (-0.17 to 0.24) | 0.7 | -0.44 (-0.67 to -0.22) | 0.0002 | -0.04 (-0.40 to 0.32) | 0.8 | -0.17 (-0.34 to -0.01) | 0.03 | -0.06 (-0.24 to 0.12) | 0.5 |
| ***Gestational hypertension*** |  |  |  |  |  |  |  |  |  |  |
| Acetate | 0.03 (-0.10 to 0.16) | 0.7 | -0.43 (-0.59 to -0.28) | 3.7x10^-8^ | -0.19 (-0.62 to 0.24) | 0.4 | -0.05 (-0.17 to 0.08) | 0.5 | 0.0 (-0.08 to 0.08) | 0.9 |
| ***Preterm birth*** |  |  |  |  |  |  |  |  |  |  |
| Glutamine | 0.56 (0.36 to 0.76) | 2.4x10^-8^ | -0.02 (-0.34 to 0.3) | 0.9 | -0.11 (-0.23 to 0.01) | 0.07 | 0.02 (-0.09 to 0.12) | 0.8 | 0.08 (-0.04 to 0.2) | 0.2 |
| Tyrosine | 0.34 (0.19 to 0.49) | 7.0x10^-6^ | -0.02 (-0.37 to 0.34) | 0.9 | -0.05 (-0.18 to 0.08) | 0.4 | 0.01 (-0.13 to 0.16) | 0.9 | -0.02 (-0.12 to 0.07) | 0.6 |
| Total lipids in very small VLDL | 0.31 (0.16 to 0.45) | 0.00004 | -0.06 (-0.34 to 0.22) | 0.7 |  |  | 0.05 (-0.04 to 0.14) | 0.3 | 0.07 (-0.04 to 0.17) | 0.2 |
| Total lipids in small LDL | 0.33 (0.16 to 0.50) | 0.0001 | -0.11 (-0.3 to 0.08) | 0.3 | 0.07 (-0.08 to 0.22) | 0.3 | 0.06 (-0.03 to 0.16) | 0.2 | 0.14 (0.04 to 0.24) | 0.004 |
| Total lipids in small HDL | -0.69 (-1.06 to -0.31) | 0.0003 | -0.06 (-0.21 to 0.08) | 0.4 |  |  | 0.07 (-0.02 to 0.17) | 0.1 | 0.05 (-0.07 to 0.17) | 0.4 |
| Total lipids in large HDL | 0.30 (0.15 to 0.45) | 0.0001 | -0.04 (-0.38 to 0.31) | 0.8 | 0.04 (-0.10 to 0.17) | 0.6 | -0.03 (-0.2 to 0.13) | 0.7 | -0.04 (-0.13 to 0.05) | 0.4 |
| Total lipids in IDL | 0.34 (0.18 to 0.5) | 0.00003 | -0.10 (-0.28 to 0.09) | 0.3 | 0.05 (-0.08 to 0.18) | 0.5 | 0.05 (-0.05 to 0.14) | 0.3 | 0.10 (0.0 to 0.20) | 0.06 |
| Glycoprotein acetyls | -0.46 (-0.62 to -0.3) | 9.0x10^-9^ | -0.06 (-0.47 to 0.34) | 0.8 | 0.01 (-0.11 to 0.14) | 0.8 | 0.10 (-0.02 to 0.22) | 0.1 | -0.01 (-0.11 to 0.09) | 0.8 |
| Total cholesterol | 0.36 (0.21 to 0.52) | 6.7x10^-6^ | -0.09 (-0.27 to 0.09) | 0.3 | 0.05 (-0.08 to 0.18) | 0.4 | 0.05 (-0.04 to 0.15) | 0.3 | 0.11 (0.01 to 0.21) | 0.04 |
| Remnant cholesterol | 0.25 (0.1 to 0.4) | 0.0009 | 0.0 (-0.19 to 0.19) | 0.9 | 0.06 (-0.16 to 0.28) | 0.6 | 0.08 (-0.01 to 0.18) | 0.08 | 0.06 (-0.03 to 0.16) | 0.2 |
| Total cholesterol in LDL | 0.29 (0.13 to 0.46) | 0.0005 | -0.12 (-0.32 to 0.07) | 0.2 | 0.05 (-0.08 to 0.18) | 0.5 | 0.06 (-0.04 to 0.15) | 0.2 | 0.14 (0.04 to 0.24) | 0.006 |
| Total cholesterol in HDL | 0.36 (0.2 to 0.52) | 1.2x10^-5^ | -0.05 (-0.36 to 0.26) | 0.7 | 0.02 (-0.15 to 0.18) | 0.9 | -0.03 (-0.17 to 0.11) | 0.7 | 0.0 (-0.09 to 0.09) | 0.9 |
| Total cholesterol in HDL2 | 0.30 (0.15 to 0.46) | 0.0001 | -0.05 (-0.36 to 0.26) | 0.7 | 0.0 (-0.19 to 0.19) | 0.9 | -0.05 (-0.17 to 0.08) | 0.5 | - | - |
| Total cholesterol in HDL3 | 0.48 (0.31 to 0.66) | 6.0x10^-8^ | -0.05 (-0.31 to 0.22) | 0.7 | 0.06 (-0.08 to 0.2) | 0.4 | 0.05 (-0.08 to 0.18) | 0.5 | - | - |
| Esterified cholesterol | 0.31 (0.15 to 0.47) | 0.0001 | -0.11 (-0.29 to 0.07) | 0.3 | 0.05 (-0.08 to 0.18) | 0.5 | 0.04 (-0.05 to 0.14) | 0.4 | 0.11 (0.01 to 0.21) | 0.03 |
| Free cholesterol | 0.49 (0.33 to 0.65) | 1.1x10^-9^ | -0.11 (-0.29 to 0.08) | 0.3 | 0.06 (-0.07 to 0.19) | 0.4 | 0.07 (-0.02 to 0.16) | 0.1 | 0.09 (-0.01 to 0.19) | 0.06 |
| Total cholines | 0.22 (0.09 to 0.35) | 0.0007 | -0.14 (-0.31 to 0.02) | 0.09 | 0.05 (-0.09 to 0.18) | 0.5 | 0.03 (-0.07 to 0.14) | 0.5 | 0.06 (-0.08 to 0.2) | 0.4 |
| Sphingomyelins | 0.39 (0.23 to 0.54) | 6.8x10^-7^ | -0.14 (-0.33 to 0.05) | 0.1 | 0.05 (-0.08 to 0.18) | 0.4 | 0.02 (-0.07 to 0.11) | 0.7 | 0.10 (0.0 to 0.20) | 0.05 |
| Apolipoprotein B | 0.25 (0.10 to 0.40) | 0.001 | -0.01 (-0.2 to 0.19) | 0.9 | 0.04 (-0.11 to 0.18) | 0.6 | 0.11 (0.01 to 0.2) | 0.02 | 0.08 (-0.02 to 0.18) | 0.1 |
| Estimated degree of unsaturation | -0.75 (-0.93 to -0.57) | 3.3x10^-16^ | -0.09 (-0.26 to 0.09) | 0.3 | -0.12 (-0.41 to 0.16) | 0.4 | -0.05 (-0.17 to 0.08) | 0.5 | 0.08 (-0.1 to 0.27) | 0.4 |
| Saturated fatty acids | 0.38 (0.19 to 0.56) | 7.3x10^-5^ | 0.02 (-0.16 to 0.19) | 0.9 | 0.08 (-0.06 to 0.22) | 0.3 | 0.08 (-0.01 to 0.17) | 0.09 | 0.01 (-0.11 to 0.13) | 0.9 |
| Glucose | -0.31 (-0.46 to -0.15) | 0.0001 | 0.14 (-0.04 to 0.32) | 0.1 | 0.07 (-0.19 to 0.33) | 0.6 | -0.02 (-0.09 to 0.06) | 0.7 | -0.05 (-0.16 to 0.06) | 0.4 |
| Citrate | 0.25 (0.12 to 0.38) | 0.0001 | 0.18 (0.01 to 0.35) | 0.03 | -0.05 (-0.18 to 0.08) | 0.4 | -0.02 (-0.12 to 0.08) | 0.7 | 0.07 (-0.04 to 0.17) | 0.2 |
| Creatinine | -0.32 (-0.45 to -0.18) | 4.5x10^-6^ | -0.1 (-0.26 to 0.07) | 0.2 | -0.27 (-0.83 to 0.28) | 0.3 | 0.07 (-0.02 to 0.17) | 0.1 | 0.06 (-0.03 to 0.14) | 0.2 |
| Albumin | -0.89 (-1.1 to -0.69) | 1.3x10^-17^ | 0.01 (-0.15 to 0.18) | 0.9 | -0.02 (-0.15 to 0.12) | 0.8 | 0.05 (-0.07 to 0.17) | 0.4 | 0.02 (-0.08 to 0.12) | 0.7 |
| Conc. of small HDL particles | -0.69 (-1.06 to -0.32) | 0.0003 | - | - | - | - | 0.07 (-0.03 to 0.17) | 0.1 | 0.05 (-0.07 to 0.16) | 0.4 |
| Conc. of very small VLDL particles | 0.25 (0.10 to 0.40) | 0.0008 | - | - | - | - | 0.07 (-0.02 to 0.16) | 0.1 | 0.07 (-0.04 to 0.17) | 0.2 |
| Conc. of IDL particles | 0.33 (0.17 to 0.49) | 0.00005 | - | - | - | - | 0.06 (-0.04 to 0.15) | 0.2 | 0.10 (0.0 to 0.20) | 0.06 |
| Conc. of small LDL particles | 0.33 (0.16 to 0.49) | 0.0001 | - | - | - | - | 0.07 (-0.02 to 0.17) | 0.1 | 0.13 (0.03 to 0.23) | 0.008 |
| Conc. of large HDL particles | 0.29 (0.14 to 0.44) | 0.0002 | - | - | - | - | -0.03 (-0.19 to 0.12) | 0.7 | -0.04 (-0.13 to 0.05) | 0.3 |
| VLDL particle size | -0.40 (-0.55 to -0.25) | 2.8x10^-7^ | 0.14 (-0.04 to 0.32) | 0.1 | 0.02 (-0.22 to 0.26) | 0.9 | 0.15 (0.04 to 0.25) | 0.006 | -0.05 (-0.15 to 0.05) | 0.3 |
| HDL particle size | 0.76 (0.59 to 0.93) | 1.4x10^-18^ | 0.0 (-0.24 to 0.25) | 0.9 | 0.03 (-0.11 to 0.17) | 0.7 | -0.06 (-0.24 to 0.11) | 0.5 | -0.03 (-0.13 to 0.06) | 0.5 |
| ***Small for gestational age*** |  |  |  |  |  |  |  |  |  |  |
| Total cholesterol in VLDL | 0.29 (0.17 to 0.42) | 4.7x10^-6^ | 0.17 (-0.03 to 0.36) | 0.09 | -0.16 (-0.47 to 0.16) | 0.3 | 0.13 (0.01 to 0.24) | 0.03 | 0.04 (-0.07 to 0.15) | 0.5 |
| Total cholesterol in HDL | -0.37 (-0.52 to -0.21) | 2.4x10^-6^ | -0.07 (-0.25 to 0.11) | 0.5 | 0.05 (-0.11 to 0.2) | 0.5 | 0.0 (-0.11 to 0.11) | 0.9 | -0.03 (-0.21 to 0.15) | 0.8 |
| Total cholesterol in HDL2 | -0.37 (-0.52 to -0.22) | 8.2x10^-7^ | -0.07 (-0.25 to 0.11) | 0.4 | 0.06 (-0.1 to 0.21) | 0.5 | -0.01 (-0.12 to 0.1) | 0.8 | -0.03 (-0.21 to 0.14) | 0.7 |
| Total cholesterol in HDL3 | -0.30 (-0.46 to -0.14) | 0.00001 | -0.05 (-0.23 to 0.14) | 0.6 | 0.02 (-0.13 to 0.18) | 0.8 | 0.05 (-0.05 to 0.16) | 0.3 | 0.02 (-0.16 to 0.19) | 0.9 |
| Total lipids in medium HDL | -0.41 (-0.56 to -0.25) | 2.6x10^-7^ | -0.02 (-0.21 to 0.17) | 0.8 | 0.25 (-0.19 to 0.68) | 0.3 | 0.07 (-0.05 to 0.18) | 0.3 | 0.01 (-0.1 to 0.12) | 0.8 |
| Total lipids in very small VLDL | 0.34 (0.20 to 0.47) | 1.4x10^-6^ | 0.15 (-0.05 to 0.35) | 0.1 | 0.01 (-0.12 to 0.15) | 0.8 | 0.17 (0.06 to 0.29) | 0.003 | 0.02 (-0.15 to 0.18) | 0.8 |
| Conc. of very small VLDL particles | 0.35 (0.21 to 0.48) | 5.2x10^-7^ | - | - | - | - | 0.17 (0.05 to 0.29) | 0.005 | 0.02 (-0.13 to 0.17) | 0.8 |
| Conc. of medium HDL particles | -0.40 (-0.55 to -0.24) | 5.0x10^-7^ | - | - | - | - | 0.07 (-0.05 to 0.19) | 0.3 | 0.01 (-0.1 to 0.12) | 0.8 |
| Alanine | 0.09 (-0.04 to 0.23) | 0.2 | 0.04 (-0.13 to 0.22) | 0.6 | -0.25 (-0.38 to -0.11) | 0.0003 | -0.07 (-0.18 to 0.04) | 0.2 | 0.09 (-0.03 to 0.21) | 0.1 |
| Histidine | -0.21 (-0.33 to -0.08) | 0.001 | 0.03 (-0.13 to 0.2) | 0.7 | -0.14 (-0.26 to -0.02) | 0.02 | 0.02 (-0.1 to 0.13) | 0.8 | 0.01 (-0.11 to 0.12) | 0.9 |
| Apolipoprotein A-I | -0.33 (-0.48 to -0.18) | 0.00002 | -0.02 (-0.19 to 0.16) | 0.8 | 0.02 (-0.14 to 0.17) | 0.8 | 0.04 (-0.07 to 0.16) | 0.4 | -0.02 (-0.2 to 0.16) | 0.8 |
| Omega-3 fatty acids | 0.34 (0.16 to 0.52) | 0.0002 | 0.0 (-0.39 to 0.38) | 0.9 | -0.04 (-0.2 to 0.11) | 0.6 | 0.11 (-0.01 to 0.24) | 0.08 | 0.04 (-0.07 to 0.15) | 0.5 |
| ***Gestational diabetes*** |  |  |  |  |  |  |  |  |  |  |
| LDL particle size | -0.25 (-0.39 to -0.10) | 0.0007 | -0.03 (-0.23 to 0.17) | 0.8 | 0.02 (-0.37 to 0.4) | 0.9 | -0.11 (-0.32 to 0.1) | 0.3 | -0.02 (-0.27 to 0.23) | 0.9 |
| Isoleucine | -0.27 (-0.41 to -0.14) | 0.00008 | -0.03 (-0.47 to 0.42) | 0.9 | -0.09 (-0.5 to 0.32) | 0.7 | -0.01 (-0.29 to 0.28) | 0.9 | -0.14 (-0.42 to 0.13) | 0.3 |
| Glucose | -0.08 (-0.23 to 0.07) | 0.3 | 0.35 (0.18 to 0.52) | 0.00005 | -0.35 (-0.79 to 0.10) | 0.1 | 0.16 (-0.58 to 0.91) | 0.7 | -0.10 (-0.30 to 0.10) | 0.3 |
| ***Large for gestational age*** |  |  |  |  |  |  |  |  |  |  |
| Valine | -0.05 (-0.22 to 0.13) | 0.6 | -0.15 (-0.31 to 0.02) | 0.09 | -0.02 (-0.12 to 0.08) | 0.7 | -0.07 (-0.14 to -0.01) | 0.02 | -0.19 (-0.29 to -0.09) | 0.0003 |
| Conc. of medium HDL particles | -0.01 (-0.25 to 0.22) | 0.9 | - | - | - | - | -0.12 (-0.18 to -0.05) | 0.0003 | -0.01 (-0.11 to 0.08) | 0.8 |
| Conc. of small HDL particles | -0.08 (-0.23 to 0.07) | 0.3 | - | - | - | - | -0.13 (-0.19 to -0.07) | 0.00006 | -0.08 (-0.16 to 0.0) | 0.1 |
| Free cholesterol in medium HDL | - | - | 0.0 (-0.21 to 0.20) | 0.9 | 0.04 (-0.24 to 0.32) | 0.8 | -0.12 (-0.18 to -0.05) | 0.0003 | -0.01 (-0.1 to 0.09) | 0.9 |
| Glycoprotein acetyls | 0.02 (-0.14 to 0.18) | 0.8 | 0.14 (-0.06 to 0.33) | 0.2 | -0.11 (-0.21 to -0.01) | 0.03 | -0.12 (-0.18 to -0.05) | 0.0007 | -0.14 (-0.38 to 0.09) | 0.2 |
| Isoleucine | 0.19 (0.04 to 0.34) | 0.01 | -0.04 (-0.21 to 0.13) | 0.6 | -0.06 (-0.16 to 0.03) | 0.2 | -0.13 (-0.2 to -0.06) | 0.0001 | -0.16 (-0.4 to 0.08) | 0.2 |
| Leucine | 0.15 (0 to 0.29) | 0.05 | -0.06 (-0.23 to 0.11) | 0.5 | -0.03 (-0.13 to 0.07) | 0.6 | -0.10 (-0.16 to -0.04) | 0.001 | -0.17 (-0.41 to 0.08) | 0.2 |
| Phospholipids in medium HDL | - | - | 0.01 (-0.21 to 0.24) | 0.9 | 0.02 (-0.26 to 0.31) | 0.9 | -0.12 (-0.18 to -0.06) | 0.0001 | -0.02 (-0.1 to 0.06) | 0.7 |
| Phospholipids in small HDL | - | - | 0.0 (-0.24 to 0.25) | 0.9 | -0.10 (-0.32 to 0.12) | 0.4 | -0.12 (-0.18 to -0.05) | 0.0003 | -0.06 (-0.2 to 0.09) | 0.4 |
| Polyunsaturated fatty acids | 0.03 (-0.21 to 0.26) | 0.8 | 0.10 (-0.07 to 0.27) | 0.3 | -0.07 (-0.17 to 0.03) | 0.2 | -0.11 (-0.17 to -0.04) | 0.001 | -0.08 (-0.32 to 0.16) | 0.5 |
| Total cholesterol in small HDL | - | - | -0.04 (-0.2 to 0.12) | 0.7 | -0.05 (-0.14 to 0.05) | 0.3 | -0.11 (-0.18 to -0.05) | 0.0008 | -0.05 (-0.13 to 0.02) | 0.2 |
| Total fatty acids | 0.06 (-0.12 to 0.24) | 0.5 | 0.1 (-0.08 to 0.27) | 0.3 | -0.10 (-0.24 to 0.04) | 0.2 | -0.11 (-0.18 to -0.05) | 0.0009 | -0.08 (-0.38 to 0.21) | 0.6 |
| Total lipids in medium HDL | -0.01 (-0.24 to 0.22) | 0.9 | 0.0 (-0.22 to 0.22) | 0.9 | 0.02 (-0.27 to 0.31) | 0.9 | -0.11 (-0.18 to -0.05) | 0.0004 | -0.01 (-0.11 to 0.09) | 0.8 |
| Total lipids in small HDL | -0.08 (-0.23 to 0.07) | 0.3 | -0.01 (-0.27 to 0.24) | 0.9 | -0.14 (-0.24 to -0.04) | 0.004 | -0.13 (-0.19 to -0.07) | 0.00006 | -0.08 (-0.16 to 0.01) | 0.1 |
| Total phosphoglycerides | 0.05 (-0.2 to 0.29) | 0.7 | 0.10 (-0.05 to 0.25) | 0.2 | 0.02 (-0.28 to 0.32) | 0.9 | -0.13 (-0.19 to -0.07) | 0.00004 | -0.01 (-0.19 to 0.18) | 1.0 |
| Total triglycerides | -0.11 (-0.29 to 0.07) | 0.2 | 0.06 (-0.13 to 0.25) | 0.5 | -0.15 (-0.24 to -0.06) | 0.001 | -0.07 (-0.14 to 0) | 0.04 | -0.11 (-0.4 to 0.19) | 0.5 |
| Triglycerides in LDL | -0.08 (-0.36 to 0.2) | 0.6 | 0.13 (-0.04 to 0.3) | 0.1 | -0.07 (-0.17 to 0.03) | 0.2 | -0.11 (-0.17 to -0.04) | 0.001 | -0.08 (-0.31 to 0.15) | 0.5 |
| Triglycerides in small LDL | - | - | 0.12 (-0.05 to 0.3) | 0.2 | -0.06 (-0.24 to 0.11) | 0.5 | -0.11 (-0.18 to -0.05) | 0.0009 | -0.11 (-0.34 to 0.12) | 0.4 |
| Triglycerides in VLDL | -0.12 (-0.25 to 0.02) | 0.09 | 0.04 (-0.15 to 0.22) | 0.7 | -0.15 (-0.24 to -0.06) | 0.001 | -0.06 (-0.13 to 0.01) | 0.1 | -0.10 (-0.38 to 0.18) | 0.5 |

Data shows the adjusted pooled mean differences in SD units (95%CIs) in NMR-derived metabolic traits for associations that reach a threshold of P≤0.001 in one of the age categories as well as the equivalent associations in all other age categories (to explore differences by age). Results are adjusted for offspring sex age, and confounders. The results in this table represent the numerical values for the results shown in Figures 1-3.
