# Supplementary material for: Effect of common pregnancy and perinatal complications on offspring metabolic traits across the life course: a multi-cohort study
Source: BMC Med. 2023 Jan 18;21:23. doi: 10.1186/s12916-022-02711-8 (PMC9850719; doi:10.1186/s12916-022-02711-8)
Supplement: Supplementary file 6 — Additional file 6: Figure S1. Predicted mean Alanine trajectories from age 7-26 years and predicted mean differences in ALSPAC offspring born small for gestational age and appropriate size for gestational age. [file 12916_2022_2711_MOESM6_ESM.docx]

| **Additional file 6: Figure S1.** Predicted mean Alanine trajectories from age 7-26 years and predicted mean differences in ALSPAC offspring born small for gestational age and appropriate size for gestational age |
| --- |
|  |
| Figure shows (a) predicted mean Alanine trajectory from age 7-26 years and (b) predicted mean difference in ALSPAC offspring born small for gestational age (SGA, N=252) and appropriate size for gestational age (AGA, N=4,480). Predicted values were obtained from adjusted (for sex and confounders) natural cubic spline mixed effects models that included an interaction term with age to allow both SGA/AGA to have different metabolic trait trajectories. |
