# Supplementary material for: Effect of common pregnancy and perinatal complications on offspring metabolic traits across the life course: a multi-cohort study
Source: BMC Med. 2023 Jan 18;21:23. doi: 10.1186/s12916-022-02711-8 (PMC9850719; doi:10.1186/s12916-022-02711-8)
Supplement: Supplementary file 7 — Additional file 7: Figure S2. Predicted mean differences in NMR-derived metabolic traits for born large for gestational age and appropriate size for gestational age offspring from the ALSPAC cohort. [file 12916_2022_2711_MOESM7_ESM.docx]

| **Additional file 7: Figure S2** Predicted mean differences in NMR-derived metabolic traits for born large for gestational age and appropriate size for gestational age offspring from the ALSPAC cohort |
| --- |
| **** |
| Figure shows the predicted mean differences in NMR-derived metabolic traits between born large for gestational age (LGA, N=500) and appropriate size for gestational age (AGA, N=4,480) offspring from the ALSPAC cohort. Predicted values were obtained from adjusted (for sex and confounders) natural cubic spline mixed effects models that included an interaction term with age to allow both LGA/AGA to have different metabolic trait trajectories. |
