# Supplementary material for: Effect of common pregnancy and perinatal complications on offspring metabolic traits across the life course: a multi-cohort study
Source: BMC Med. 2023 Jan 18;21:23. doi: 10.1186/s12916-022-02711-8 (PMC9850719; doi:10.1186/s12916-022-02711-8)
Supplement: Supplementary file 8 — Additional file 8: Table S3. Cohort-specific acknowledgements and funding statements. [file 12916_2022_2711_MOESM8_ESM.docx]

# **Additional file 8: Table S3** Cohort-specific acknowledgements and funding statements

| **1. Avon Longitudinal Study of Parents and Children (ALSPAC)**  We are extremely grateful to all the families who took part in this study, the midwives for their help in recruiting them, and the whole ALSPAC team, which includes interviewers, computer and laboratory technicians, clerical workers, research scientists, volunteers, managers, receptionists and nurses. The UK Medical Research Council and Wellcome (Grant ref: 217065/Z/19/Z) and the University of Bristol provide core support for ALSPAC. A comprehensive list of grant funding is available on the ALSPAC website (<http://www.bristol.ac.uk/alspac/external/documents/grant-acknowledgements.pdf>).  Study data were collected and managed using REDCap electronic data capture tools hosted at the University of Bristol.1 REDCap (Research Electronic Data Capture) is a secure, web-based software platform designed to support data capture for research studies. |
| --- |
| **2. Born in Bradford Study (BiB)**  BiB receives core funding from the Wellcome Trust (WT101597MA and 223601/Z/21/Z), a joint grant from the UK Medical and Economic and Social Science Research Councils (MR/N024397/1), British Heart Foundation (CS/16/4/32482), and the National Institute of Health Research under its Applied Research Collaboration for Yorkshire and Humber (NIHR200166) and Clinical Research Network research delivery support. BiB is only possible because of the enthusiasm and commitment of the Children and Parents in BiB. We are grateful to all the participants, teachers, school staff, health professionals and researchers who have made BiB happen |
| **3. Young Finns Study (YFS)**  The Young Finns Study has been financially supported by the Academy of Finland: grants 322098, 286284, 134309 (Eye), 126925, 121584, 124282, 129378 (Salve), 117787 (Gendi), and 41071 (Skidi); the Social Insurance Institution of Finland; Competitive State Research Financing of the Expert Responsibility area of Kuopio, Tampere and Turku University Hospitals (grant X51001); Juho Vainio Foundation; Paavo Nurmi Foundation; Finnish Foundation for Cardiovascular Research ; Finnish Cultural Foundation; The Sigrid Juselius Foundation; Tampere Tuberculosis Foundation; Emil Aaltonen Foundation; Yrjö Jahnsson Foundation; Signe and Ane Gyllenberg Foundation; Diabetes Research Foundation of Finnish Diabetes Association; This project has received funding from the European Union’s Horizon 2020 research and innovation programme under grant agreements No 848146 (To Aition) and No 755320 (TAXINOMISIS); This project has received funding from the European Research Council (ERC) advanced grants under grant agreement No 742927 (MULTIEPIGEN project); Tampere University Hospital Supporting Foundation and Finnish Society of Clinical Chemistry, and the Cancer Foundation Finland. |
| **4. Northern Finland Birth Cohorts 1986 and 1966 (NFBC1986 and NFBC1966)**  The authors are very grateful to all the participants who took part in the Northern Finland Birth Cohort 1966 and 1986 studies; to the whole study teams, including research staff and all others involved in data collection and processing; and to those in the oversight and management of the studies. NFBC1986 received funding from EU QLG1-CT-2000-01643 (EUROBLCS) Grant no. E51560, NorFA Grant no. 731, 20056, 30167, USA / NIH 2000 G DF682 Grant no. 50945. NFBC1966 31y follow-up received funding from University of Oulu Grant no. 65354, Oulu University Hospital Grant no. 2/97, 8/97, Ministry of Health and Social Affairs Grant no. 23/251/97, 160/97, 190/97, National Institute for Health and Welfare, Helsinki Grant no. 54121, Regional Institute of Occupational Health, Oulu, Finland Grant no. 50621, 54231. NFBC1966 46y follow-up received funding from University of Oulu Grant no. 24000692, Oulu University Hospital Grant no. 24301140, ERDF European Regional Development Fund Grant no. 539/2010 A31592. |
| **5. Helsinki Birth Cohort Study (HBCS)**  HBCS was supported by Emil Aaltonen Foundation; Finnish Foundation for Diabetes Research; Foundation for Pediatric Research, Novo Nordisk Foundation; Signe and Ane Gyllenberg Foundation; Sigrid Jusélius Foundation; Samfundet Folkhälsan; Finska Läkaresällskapet; Liv och Hälsa; the Academy of Finland supported (grant no. 129369, 129907, 135072, 129255, and 126775); European Commission within the 7th Framework Programme (DORIAN, grant agreement no. 278603); and European Union Horizon 2020 programme (DYNAHEALTH grant no. 633595). |
| **6. Barwon Infant Study (BIS)**  We thank the BIS participants for the generous contribution they have made to this project. We also thank current and past staff for their efforts in recruiting and maintaining the cohort and in obtaining and processing the data and biospecimens. The establishment work and infrastructure for the BIS was provided by the Murdoch Children’s Research Institute, Deakin University and Barwon Health. Subsequent funding was secured from the National Health and Medical Research Council of Australia, The Jack Brockhoff Foundation, the Scobie Trust, the Shane O’Brien Memorial Asthma Foundation, the Our Women’s Our Children’s Fund Raising Committee Barwon Health, The Shepherd Foundation, the Rotary Club of Geelong, the Ilhan Food Allergy Foundation, GMHBA Limited and the Percy Baxter Charitable Trust, Perpetual Trustees. In-kind support was provided by the Cotton On Foundation and CreativeForce. Research at Murdoch Children’s Research Institute is supported by the Victorian Government's Operational Infrastructure Support Program. This work was also supported by NHMRC Senior Research Fellowships (1064629 to DB; 1045161 to RS) and NHMRC Investigator Grants to DB (1175744). |
| **7. Longitudinal Study of Australian Children’s Child Health CheckPoint (CheckPoint)**  This paper uses unit record data from Growing Up in Australia, the Longitudinal Study of Australian Children. The study is conducted in partnership between the Department of Social Services (DSS), the Australian Institute of Family Studies (AIFS) and the Australian Bureau of Statistics (ABS). The CheckPoint work was supported by the National Health and Medical Research Council (NHMRC) of Australia [1041352, 1109355]; the Royal Children’s Hospital Foundation [2014-241]; the Murdoch Children’s Research Institute (MCRI); The University of Melbourne, the National Heart Foundation of Australia [100660]; Financial Markets Foundation for Children [2014-055, 2016-310]; the Victoria Deaf Education Institute; and MBIE Catalyst grant (The New Zealand-Australia Life Course Collaboration on Genes, Environment, Nutrition and Obesity (GENO); UOAX1611; to JOS). This work was also supported by NHMRC Senior Research Fellowships (1046518 to MW; 1064629 to DB; 1045161 to RS) and NHMRC Investigator Grants to DB (1175744). Research at the MCRI is supported by the Victorian Government's Operational Infrastructure Support Program. REDCap (Research Electronic Data Capture) electronic data capture tools were used in this study. More information about this software can be found at: [www.projectredcap.org](http://www.projectredcap.org). The authors thank the LSAC and CheckPoint study participants, staff and students for their contributions. |
| **8. Generation R Study**  The authors gratefully acknowledge the contribution of participants, research collaborators, general practitioners, hospitals, midwives, and pharmacies in Rotterdam. The general design of the Generation R Study is made possible by financial support from the Erasmus MC, University Medical Center, Rotterdam, Erasmus University Rotterdam, Netherlands Organization for Health Research and Development (ZonMw), Netherlands Organisation for Scientific Research (NWO), Ministry of Health, Welfare and Sport and Ministry of Youth and Families. This project received funding from the European Union's Horizon 2020 research and innovation programme (LIFECYCLE, grant agreement No 733206, 2016, EUCANConnect grant agreement No 824989; ATHLETE, grant agreement No 874583, and under the ERA-NET Cofund action (no 727565), European Joint Programming Initiative “A Healthy Diet for a Healthy Life” (JPI HDHL, EndObesity, ZonMW the Netherlands, (no. 529051026)). VWVJ received a European Research Council Consolidator Grant (ERC-2014-CoG-648916). RG received funding of the Dutch Heart Foundation (grant number 2017T013), the Dutch Diabetes Foundation (grant number 2017.81.002), and the Netherlands Organization for Health Research and Development (NWO, ZonMW, grant number 543003109). The study sponsors had no role in the study design, data analysis, interpretation of data, or writing of this report. |
